# Degradation Effect and Magnetoelectric Transport Properties in CrBr_3_ Devices

**DOI:** 10.3390/ma15093007

**Published:** 2022-04-21

**Authors:** Yanfei Wu, Mengyuan Zhu, Ruijie Zhao, Xinjie Liu, Jianxin Shen, He Huang, Shipeng Shen, Liyuan Zhang, Jingyan Zhang, Xinqi Zheng, Shouguo Wang

**Affiliations:** 1 Beijing Advanced Innovation Center for Materials Genome Engineering, School of Materials Science and Engineering, University of Science and Technology Beijing, Beijing 100083, China; yanfeiwu@ustb.edu.cn (Y.W.); myzhu@xs.ustb.edu.cn (M.Z.); rjzhao@xs.ustb.edu.cn (R.Z.); liuxinjie@xs.ustb.edu.cn (X.L.); jxshen@ustb.edu.cn (J.S.); hhuang@ustb.edu.cn (H.H.); m202120491@xsustb.cn (L.Z.); jyzhang@ustb.edu.cn (J.Z.); zhengxq@ustb.edu.cn (X.Z.); 2 Institute of Advanced Materials, Beijing Normal University, Beijing 100875, China; shenshipeng@bnu.edu.cn

**Keywords:** CrBr_3_, 2D magnetic materials, degradation, magnetoelectric transport

## Abstract

Two-dimensional (2D) magnetic materials exhibiting unique 2D-limit magnetism have attracted great attention due to their potential applications in ultrathin spintronic devices. These 2D magnetic materials and their heterostructures provide a unique platform for exploring physical effect and exotic phenomena. However, the degradation of most 2D magnetic materials at ambient conditions has so far hindered their characterization and integration into ultrathin devices. Furthermore, the effect of degradation on magnetoelectric transport properties, which is measured for the demonstration of exotic phenomena and device performance, has remained unexplored. Here, the first experimental investigation of the degradation of CrBr_3_ flakes and its effect on magnetoelectric transport behavior in devices is reported. The extra magnetic compounds derived from oxidation-related degradation play a significant role in the magnetoelectric transport in CrBr_3_ devices, greatly affecting the magnetoresistance and conductivity. This work has important implications for studies concerning 2D magnetic materials measured, stored, and integrated into devices at ambient conditions.

## 1. Introduction

Two-dimensional (2D) intrinsic magnetic materials (also known as 2D magnetic materials or 2D magnets) have attracted considerable attention since the discovery of unique 2D-limit magnetism in ${\mathrm{CrI}}_{3}$ and ${\mathrm{Cr}}_{2}{\mathrm{Ge}}_{2}{\mathrm{Te}}_{6}$ in 2017 [1,2]. So far, abundant 2D magnetic materials [2,3,4,5], such as Fe_3_GeTe_2_, ${\mathrm{RuCl}}_{3}$, ${\mathrm{CrX}}_{3\;}\left(X=I,\;\mathrm{Br},\;\mathrm{Cl}\right)$, ${\mathrm{CrXTe}}_{3\;}\left(X=\mathrm{Ge},\;\mathrm{Si}\right)$, ${\mathrm{CrX}}_{2\;}\left(X=\mathrm{Se},\;\mathrm{Te},\;S\right)$, ${\mathrm{MnX}}_{2\;}\left(X=\mathrm{Se},\;S\right)$, ${\mathrm{VX}}_{3\;}\left(X=I,\;\mathrm{Br}\right)$, and ${\mathrm{XPS}}_{3\;}\left(X=\mathrm{Mn},\;\mathrm{Fe},\;\mathrm{Ni}\right)$, have been discovered, and they cover a variety of electrical properties, including metals, semimetals, semiconductors, and insulators. Rich magnetic properties [1,3,6,7], such as out-of-plane or in-plane magnetic anisotropy in 2D limit, ferromagnetic coupling or antiferromagnetic coupling between layers, and thickness-dependent and electrostatically tunable magnetic behavior, have been reported in these materials. The combination of 2D magnetic materials with different properties has led to the creation of unique heterostructures, together with the investigation of theoretically expected or novel physical phenomena, and the development of ultrathin spintronic devices [3,4,5,8,9,10,11,12].

However, most 2D magnetic materials suffer from degradation in the ambient environment, which originates from the sensitivity to $O_{2}$ and $H_{2}O$ [13,14,15]. There are also photocatalytic degradation and photochemical or photothermal oxidation-related degradation in the presence of oxygen and moisture, closely depending on various factors such as light exposure and temperature [13,16]. For chromium trihalides (${\mathrm{CrX}}_{3},\;X=I,\;\mathrm{Br},\;\mathrm{Cl}$), chromium bromide (${\mathrm{CrBr}}_{3}$) and chromium chloride (${\mathrm{CrCl}}_{3}$) are more stable than chromium triiodide (${\mathrm{CrI}}_{3}$), and this condition is related to the stability of lone-pair electrons of the halogen element (X) atoms that terminate the surfaces of layers. ${\mathrm{CrI}}_{3}$ shows the first visible signs of degradation within seconds under air and light conditions under an optical microscope [13]. It slowly degrades in ambient environment with the coverage of ${\mathrm{Al}}_{2}O_{3}$, PMMA, or h-BN. This result also depends on light exposure and temperature, of which the former is related to the photocatalytic substitution of iodine by water. The fragility of 2D magnetic materials to ambient conditions hinders their characterization and integration into ultrathin devices that are functionalized by interfacial effect, spin-orbit torque-driven magnetization switching, exotic phenomena, and so on [3,10,11,17]. Some studies have focused on avoiding the degradation or improving the air stability of 2D magnetic materials in ambient environment [18,19,20,21,22,23]. Generally, to avoid or reduce degradation, the exfoliation of these 2D magnetic materials and their heterostructures stacking process are carried out in a glove box under inert atmosphere. Whole 2D magnetic material-based devices can be encapsulated by hexagonal boron nitride (h-BN) before being taken out. As a typical example, graphene/${\mathrm{CrI}}_{3}$/graphene spin-filter magnetic tunnel junctions were successfully fabricated by encapsulating h-BN, and they showed a tunneling magnetoresistance ratio of 19,000% [23]. Encapsulation of 2D materials with exfoliated h-BN single crystal flakes has been widely used in 2D material research [8,12,18,23]. However, 2D magnetic materials that are deposited or exfoliated on ${\mathrm{SiO}}_{2}/\mathrm{Si}$ substrates still degrade when covered by ${\mathrm{Al}}_{2}O_{3}$, PMMA, or h-BN capping films [13], due to moisture and oxygen leakage through the capping film/${\mathrm{SiO}}_{2}$ and the 2D magnetic material/${\mathrm{SiO}}_{2}$ interfaces. In addition, great efforts have been devoted to improving the air stability of 2D magnetic materials from synthesis and modification strategies [14,21,22,24], such as atomic layer deposition passivation [21], passivation from air oxidation by thiol molecules [24], and growing air-stable ${\mathrm{CrSe}}_{2}$ nanosheets on a dangling-bond-free ${\mathrm{WSe}}_{2}$ substrate [22].

Understanding the stability of 2D magnetic materials, the degradation effect on device performance, and the derived physical phenomena at ambient conditions is crucial for their characterization and applications in ambient conditions. Thus far, a limited number of studies have investigated the degradation effect of 2D magnetic materials on device performance and the derived physical phenomena [25]. Furthermore, the effect of degradation on magnetoelectric transport behavior (such as magnetoresistance), which is usually measured in a cryogenic transport measurement system for demonstrating exotic phenomena and device performance, remains unexplored.

In this work, the degradation of ${\mathrm{CrBr}}_{3}$, a ferromagnetic semiconductor 2D material that has been extensively studied recently, was investigated in an ambient environment by optical microscopy. The X-ray photoelectron spectroscopy (XPS) spectra demonstrated extra compounds derived from oxidation-related degradation on the ${\mathrm{CrBr}}_{3}$ surface. Furthermore, the degradation effect on magnetoelectric transport behavior in ${\mathrm{CrBr}}_{3}$ devices was investigated by the analysis of temperature-dependent Hall magnetoresistance and conductivity in ${\mathrm{CrBr}}_{3}$ devices.

## 2. Materials and Methods

### 2.1. Device Fabrication

The ${\mathrm{CrBr}}_{3}$ crystals were synthesized by the chemical vapor transport (CVT) method using elemental precursors of chromium (Cr, Alfa Aesar 99%) and tellurium bromide (${\mathrm{TeBr}}_{4}$, Alfa Aesar 99.9%) [26]. Pure crystals of ${\mathrm{CrBr}}_{3}$ were obtained by mixing Cr powder and ${\mathrm{TeBr}}_{4}$ with a molar ratio of 1:1.5 and then annealing at 750 °C for 72 h. The nanometer-thick ${\mathrm{CrBr}}_{3}$ flakes were mechanically exfoliated from ${\mathrm{CrBr}}_{3}$ crystals onto 285 nm thick ${\mathrm{SiO}}_{2}/\mathrm{Si}$ substrates. The pre-patterned Pt (6 nm) electrodes on ${\mathrm{SiO}}_{2}/\mathrm{Si}$ substrates were fabricated through micro–nano fabrication processes. Finally, two types of ${\mathrm{CrBr}}_{3}$ devices were prepared by transferring ${\mathrm{CrBr}}_{3}$ flake onto pre-patterned Pt electrodes using a poly(bisphenol A carbonate) (PC)/polydimethylsiloxane (PDMS) polymer-based transfer method [27].

### 2.2. Characterization and Measurements

X-ray photoelectron spectroscopy (XPS) data were acquired on PHI Quantera II XPS spectrometer employing Al Kα radiation. The XPS binding energies were calibrated using the C 1*s* peak (284.5 eV). Magnetic measurements of ${\mathrm{CrBr}}_{3}$ bulk crystals and temperature-dependent magnetoelectric transport measurements of ${\mathrm{CrBr}}_{3}$ devices were performed in a physical property measurement system (PPMS DynaCool, Quantum Design). The Hall magnetoresistance was obtained using DC measurement and AC measurement, in order to confirm the repeatability of data. For AC measurements by the lock-in technique, the AC current was provided by the Keithley 6221 current source, and the voltage signal was measured by Model SR830 DSP lock-in amplifiers. For DC measurements, the Keithley 2400 SourceMeter and Keithley 2182A Nanovoltmeter were used to provide the current and to detect the voltage, respectively.

## 3. Results and Discussion

**Figure 1:**
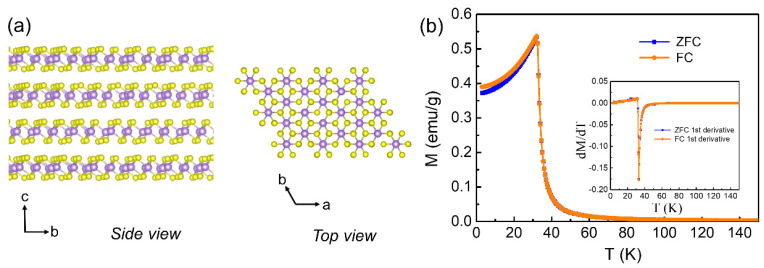
(**a**) Crystal structure of ${\mathrm{CrBr}}_{3}$ below the Curie temperature. Left: side view of *bc* plane. Right: top view of the *ab* plane. The colored balls represent Cr (purple) and Br (yellow), respectively. (**b**) Temperature dependence of ZFC and FC magnetization for a CrBr_3_ platelet under an in-plane magnetic field of 50 mT. Inset: the first-order temperature derivatives of ZFC and FC magnetization.

${\mathrm{CrBr}}_{3}$ is a layered ferromagnetic semiconductor with out-of-plane magnetic order below the Curie temperature ($T_{C}$). ${\mathrm{CrBr}}_{3}$ crystallizes in a lamellar structure with trigonal space group $R\bar{3}$ (148), in which ${\mathrm{Cr}}^{3+}$ ions arrange in the honeycomb magnetic lattice and are surrounded by edge-sharing octahedra formed by ${\mathrm{Br}}^{-}$ ions [28,29]. As shown in Figure 1a, the Br–Cr–Br layers are stacked along the *c*-axis and stacked together via van der Waals force. The magnetic moments defined by Cr layers are oriented perpendicular to the *ab* plane and aligned ferromagnetically within each layer, and the layers are also ferromagnetically coupled. The magnetic properties of a ${\mathrm{CrBr}}_{3}$ platelet were characterized by a vibrating sample magnetometer in PPMS. As shown in Figure 1b, zero-field-cooled (ZFC) and field-cooled (FC) magnetization and their first derivatives exhibited a paramagnetic–ferromagnetic phase transition at $T_{C}$ = 33 K, nearly close to the $T_{C}$ obtained by temperature-dependent M-H curves (Figure S1), and it is also in good accordance with the $T_{C}$ = 32–40 K of ${\mathrm{CrBr}}_{3}$ observed in previous studies [28,29,30].

**Figure 2:**
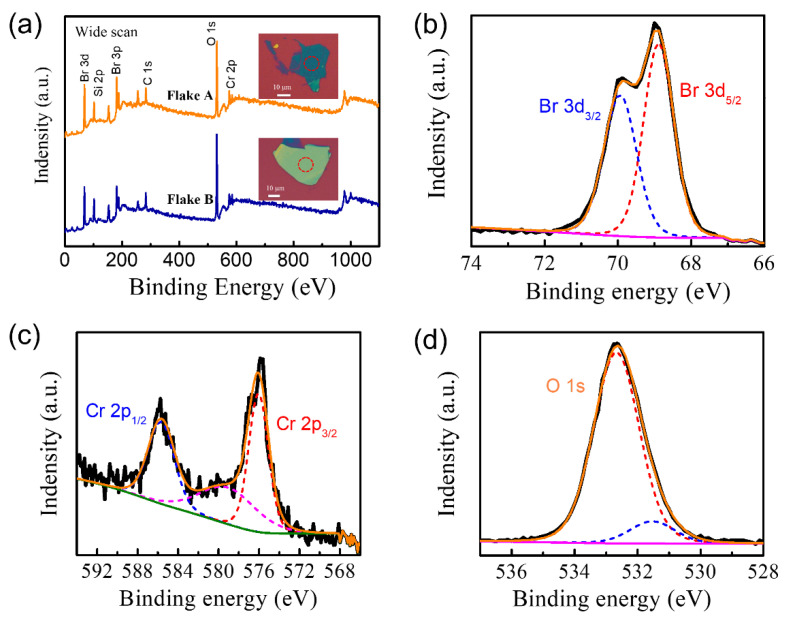
XPS spectra of CrBr_3_ flakes exfoliated on SiO_2_/Si substrates. (**a**) A wide scan of two CrBr_3_ flakes. Inset: the optical images of CrBr_3_ flakes. (**b**–**d**) The fine scans of Br 3*d*, Cr 2*p*, and O 1*s* of flake A, respectively. The dotted lines indicate the deconvoluted curves of Br 3*d*, Cr 2*p*, and O 1*s* spectra. The two red circles in (**a**) indicate the location of the respective XPS measurements.

The chemical composition of ${\mathrm{CrBr}}_{3}$ flakes on ${\mathrm{SiO}}_{2}/\mathrm{Si}$ substrates was analyzed by XPS. The ${\mathrm{CrBr}}_{3}$ flakes were mechanically exfoliated on ${\mathrm{SiO}}_{2}/\mathrm{Si}$ substrates in the air. Figure 2a shows the XPS spectra of two ${\mathrm{CrBr}}_{3}$ flakes on ${\mathrm{SiO}}_{2}/\mathrm{Si}$ substrates, and the inset presents the optical images of the corresponding ${\mathrm{CrBr}}_{3}$ flakes. The expected elemental peaks of Cr 2*p*, Br 3*d*, Si 2*p*, C 1*s*, and O 1*s* were observed quantitatively. The ${\mathrm{SiO}}_{2}/\mathrm{Si}$ substrate contributed to Si 2*p* and O 1*s* peaks. For ${\mathrm{CrBr}}_{3}$ flakes, the binding energies of the Br 3*d*_5/2_ and Br 3*d*_3/2_ core level were located at 68.9 and 69.9 eV, respectively (Figure 2b). In addition, the two peaks located at 576.1 and 585.7 eV corresponded to Cr 2*p*_3/2_ and Cr 2*p*_1/2_ (Figure 2c), respectively. However, an additional peak at 579.1 eV could be seen, most likely related to chromium oxides (such as ${\mathrm{CrO}}_{2}$ and ${\mathrm{Cr}}_{2}O_{3}$) [31]. This additional Cr 2*p*_3/2_ peak centered at 579.1 eV could be fitted to obtain a large FWHW of 6.6 eV, associated with the mixture of chromium oxides and other compounds. In addition, the main features of the XPS spectra were identical with other ${\mathrm{CrBr}}_{3}$ flakes on ${\mathrm{SiO}}_{2}/\mathrm{Si}$ substrates but differed in the content of additional compounds. Our XPS results are in good agreement with the oxidation-related degradation resulting in the formation of chromium oxide [32].

**Figure 3:**
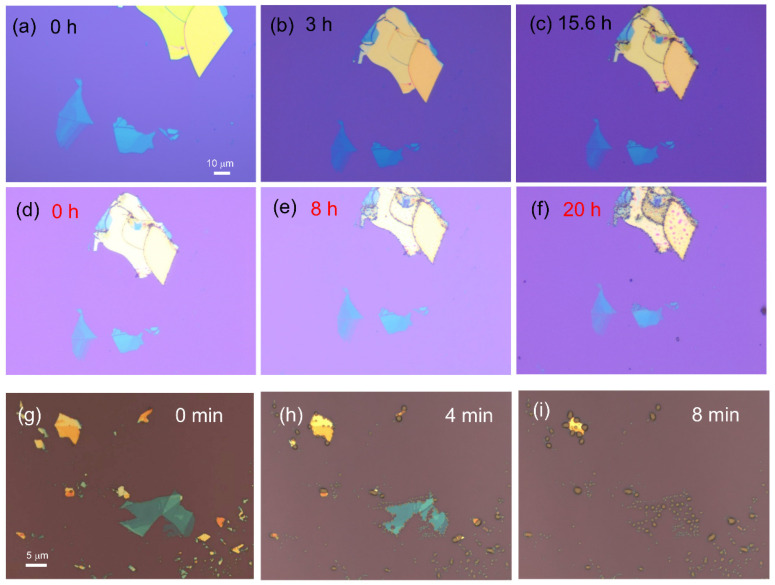
Optical microscopy images of (**a**–**c**) CrBr_3_ flakes placed in ambient atmosphere for 0 h, 3 h, and 15.6 h, respectively. (**d**–**f**) The CrBr_3_ flakes exposed to light irradiation for 0 h, 8 h, and 20 h after storage in ambient atmosphere for 3 days. (**g**–**i**) The CrBr_3_ flakes exposed to stronger light irradiation in ambient atmosphere for 0 min, 4 min, and 8 min, respectively.

The XPS results indicate the extra compounds derived from degradation on the ${\mathrm{CrBr}}_{3}$ surface, although no obvious degradation could be observed under optical microscopy after storage in ambient atmosphere for 2 days. Furthermore, the degradation process of ${\mathrm{CrBr}}_{3}$ flakes on the ${\mathrm{SiO}}_{2}/\mathrm{Si}$ substrate was monitored by optical microscopy in ambient atmosphere (Figure 3). The degradation of ${\mathrm{CrBr}}_{3}$ flakes was slow in ambient atmosphere, and they showed no significant change after storage in ambient atmosphere for 15.6 h (Figure 3a–c). Subsequently, dark-gray bubbles or droplets cluttered on the edges, and the surfaces of flakes gradually increased under light irradiation (Figure 3d–f). This irradiation was directly provided by the light emitted from the 50× objective of an optical microscope during the whole measurement. The above phenomena demonstrate that the degradation of ${\mathrm{CrBr}}_{3}$ flakes was moderate but became faster under light exposure. Furthermore, the degradation was accelerated by a reduction in flake thickness (Figure 3 and Figure S2 in Supplementary Materials). The degradation process for the ${\mathrm{CrBr}}_{3}$ flakes with different thicknesses were also monitored using another optical microscope that could provide a stronger light irradiation from a 100× objective. As shown in Figure 3g–i, some tiny and gray droplets mainly formed at the flake edges and grain boundaries, growing rapidly under stronger light irradiation. Then, the droplets merged into larger ones, which spread from the flake edges and finally covered the majority of the flake surface until the flakes were completely decomposed. A short decomposition time of several minutes was observed due to the stronger light irradiation and higher temperature.

Oxygen, humidity, and light exposure are crucial factors that affect the air stability of 2D magnetic materials [13,14,15]. Moreover, the size, thickness, starting condition, and quality of each flake, even the substrate, play a particular role in the degradation, which are difficult to be quantitatively evaluated in ambient atmosphere. The observation of scanning electron microscope images further confirmed that defects, such as grain boundaries, present a dominant contribution to material degradation, acting as the sources of inducement and propagation [25]. This condition can be used to explain where the bubbles or droplets appear firstly. 

A tentative explanation for degradation is the reaction with ambient $O_{2}$ and $H_{2}O$, as well as the subsequent formation of intermediate chromium oxide bromides, chromium oxides or aqua chromium halide [13,32]. According to previous studies of ${\mathrm{CrI}}_{3}$ [13], water reacts with ${\mathrm{CrBr}}_{3}$ to possibly form partially hydrated ${\mathrm{CrBr}}_{3-x}{\left(H_{2}O\right)}_{x}^{x+}$ under ambient conditions, and the reactivity can be accelerated by light irradiation. Thus, the gray droplets on the ${\mathrm{CrBr}}_{3}$ flakes shown in Figure 3 and Figure S2 are related to photocatalytic degradation, in which the main degradation pathway is the photocatalytic substitution of bromine by water. In addition, $O_{2}$ can react with ${\mathrm{CrBr}}_{3}$ to form chromium oxides at high temperatures, similar to the case of CrI_3_ where degradation causes the formation of ${\mathrm{Cr}}_{2}O_{3}$ at 180 °C, resulting in teal-colored ${\mathrm{CrI}}_{3}$ thin flakes [13].

**Figure 4:**
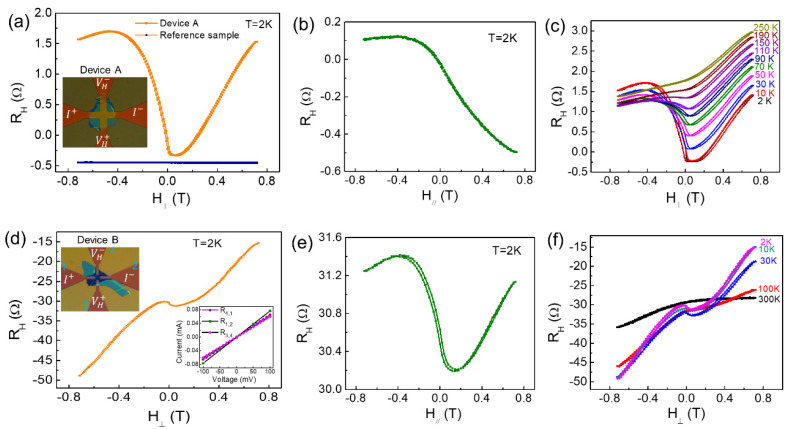
(**a**,**b**) Hall magnetoresistance of device A consisting of a CrBr_3_ flake on Pt Hall bar electrodes under out-of-plane and in-plane magnetic fields, respectively. The Pt Hall bar device was used as the reference sample. The CrBr_3_ film is shown in the teal-colored area. (**c**) Temperature dependence of Hall magnetoresistance of device A. (**d**,**e**) Hall magnetoresistance of device B consisting of a CrBr_3_ flake on of Pt Hall bar electrodes without a crossing area. Inset of (**d**): linear I–V curves of CrBr_3_ flake. The numbers 1, 2, 3, 4 are used to mark four electrode ports. (**f**) Temperature dependence of Hall magnetoresistance of device B.

To investigate the degradation effect on the magnetoelectric transport properties of ${\mathrm{CrBr}}_{3}$ and its heterostructure devices, two kinds of ${\mathrm{CrBr}}_{3}$ devices were fabricated, namely, devices A and B. Device A was fabricated by transferring a ${\mathrm{CrBr}}_{3}$ flake on pre-patterned Pt Hall bar electrodes (inset in Figure 4a). Device B was fabricated by transferring a ${\mathrm{CrBr}}_{3}$ flake on Pt Hall bar electrodes without a crossing area (inset in Figure 4d). Device A could be used to study the magnetic proximity effect, spin Hall magnetoresistance, and current-induced magnetization switching in the ${\mathrm{CrBr}}_{3}$/Pt heterostructure. As shown in Figure 4a,b, device A showed a remarkable Hall magnetoresistance ($R_{H}$) curve under out-of-plane magnetic fields ($H_{⊥}$) and in-plane magnetic fields ($H_{//}$), similar to the ferromagnetic behavior, which was notably larger than that for the reference sample (only Pt Hall bar device). Figure 4c shows the temperature dependence of $R_{H}$ in device A. The ferromagnetic-like behavior persisted at 110 K, which is greatly higher than the $T_{C}$ of 32–40 K for ${\mathrm{CrBr}}_{3}$. Therefore, the origin of the anomalous Hall effect (AHE) in semiconductor ${\mathrm{CrBr}}_{3}$ film or magnetized Pt film can be excluded because the production of a remarkable R_H_ above Tc is impossible. The R_H_ in device A was most likely produced by the AHE of extra magnetic compounds derived from the degradation of ${\mathrm{CrBr}}_{3}$. To further clarify the origin of R_H_ in device A, the R_H_ of the ${\mathrm{CrBr}}_{3}$ flake on device B was measured. As shown in Figure 4d,e, device B featured R_H_ curves under $H_{⊥}$ and $H_{//}$. As shown in Figure 4d, the R_H_ change of ~1 Ω was obtained when $H_{⊥}$ changed from +0.04 T to −0.04 T, and the R_H_ curve contained nearly linear magnetoresistance under positive and negative $H_{⊥}$. It is worth pointing out that linear I–V curves of ${\mathrm{CrBr}}_{3}$ flakes were observed in device B (inset in Figure 4d), indicating the metallic characteristic of the “${\mathrm{CrBr}}_{3}$ flake”. In addition, the same R_H_ curve was observed for device A under $H_{⊥}$ and device B under $H_{//}$, (Figure 4a,e). Figure 4f shows the R_H_ curve maintained at 100 K, which is greatly higher than the $T_{C}$ of ${\mathrm{CrBr}}_{3}$. These results on device B further confirmed the extra magnetic compounds derived from the degradation of the ${\mathrm{CrBr}}_{3}$ flake. However, determining specific magnetic compounds from these magnetoelectric measurements is difficult due to multiple compounds included in the degraded ${\mathrm{CrBr}}_{3}$. Furthermore, degradation can be judged by the conductivity or resistance of the ${\mathrm{CrBr}}_{3}$ flake. In device A, the resistance of the ${\mathrm{CrBr}}_{3}$ flake was about 4662 Ω according to the I–V curves and parallel resistance formula $\frac{R_{A}+R_{Pt}}{R_{A}\cdot R_{Pt}}$. In device B, the resistance of the ${\mathrm{CrBr}}_{3}$ flake was in the range of 1000–2000 Ω according to the I–V curves (inset in Figure 4d). Thus, there was a resistance of ~$10^{3\;}$Ω for extra magnetic compounds derived from degradation.

The ${\mathrm{CrBr}}_{3}$ flakes used for devices A and B were stored for 6 and 2 months in a low- vacuum environment (~$10^{-1}$ Pa), respectively, before transferring to pre-patterned electrodes. The above magnetoelectric measurement results confirmed that teal-colored ${\mathrm{CrBr}}_{3}$ thin flakes without droplets (insets in Figure 4a,d) underwent oxidation-related degradation in which the main degradation pathway was thermocatalytic conversion to chromium oxides in an atmosphere with $O_{2}$. The degradation occurred mainly during the transfer process, in which the polymer-based transfer medium was melted on the ${\mathrm{CrBr}}_{3}$ flake at 155 °C. The magnetoelectric measurement results agree well with the above XPS results, indicating the formation of chromium oxides on ${\mathrm{CrBr}}_{3}$ thin flakes without droplets. Therefore, in addition to the absence of oxygen and moisture, high-temperature treatment should be avoided while fabricating devices based on air-sensitive 2D magnetic materials, to minimize the influence of degradation on performance and the derived physical phenomena in these devices.

**Figure 5:**
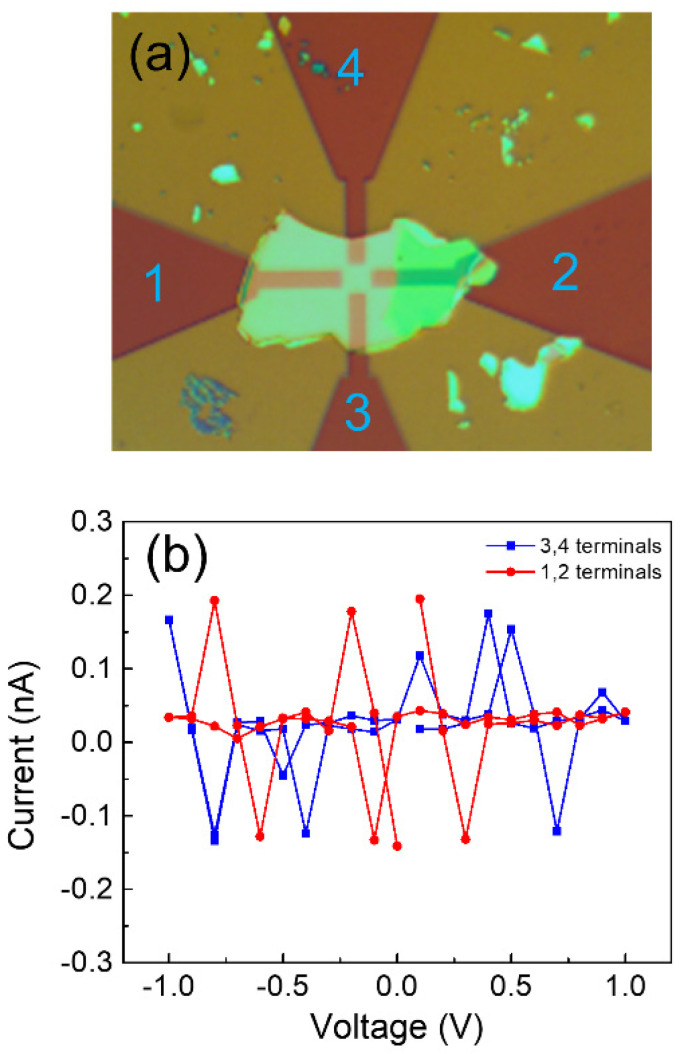
(**a**) Optical microscopy image of device C consisting of a fresh and thick CrBr_3_ flake on Pt Hall bar electrodes without a crossing area. The thick CrBr_3_ flake is shown in the light yellow area; (**b**) I–V curves of the CrBr_3_ flake in device C. The numbers 1, 2, 3, 4 are used to mark four electrode ports.

For the ${\mathrm{CrBr}}_{3}$ flakes without degradation, the R_H_ and linear I–V curves were difficult to obtain due to the semiconductor characteristics. To minimize the degradation, we exfoliated the thicker ${\mathrm{CrBr}}_{3}$ flakes and immediately transferred them onto the Pt Hall bar electrodes for measurements. Device C was prepared by transferring a fresh and thicker ${\mathrm{CrBr}}_{3}$ flake on Pt Hall bar electrodes without a crossing area (Figure 5a). No linear I–V curve was obtained for device C (Figure 5b), indicating the poor electrical conductivity of semiconductor ${\mathrm{CrBr}}_{3}$ without considerable degradation. 

Our study demonstrates that extra magnetic compounds mainly derived from oxidation-related degradation significantly affected the magnetoresistance and conductivity of ${\mathrm{CrBr}}_{3}$ devices, revealing the non-negligible effect of degradation on the magnetoelectric transport behavior of ${\mathrm{CrBr}}_{3}$ thin flakes and their heterostructure devices. For bulk platelets, surface degradation showed a remarkable effect on the magnetic properties of bulk ${\mathrm{VI}}_{3}$ [25]. Therefore, understanding the degradation process and its outcomes on magnetoelectric transport behavior in our study is necessary for the interpretation of the experimental results for functional devices based on air-sensitive 2D magnetic materials.

## 4. Conclusions

In summary, ${\mathrm{CrBr}}_{3}$ devices were successfully fabricated to investigate the degradation effect of ${\mathrm{CrBr}}_{3}$ flakes on the magnetoelectric transport properties. The extra compounds derived from oxidation-related degradation in ${\mathrm{CrBr}}_{3}$ were confirmed by XPS spectra, significantly affecting the magnetoresistance and conductivity in ${\mathrm{CrBr}}_{3}$ devices with remarkable Hall magnetoresistance and the conductive characteristic of the degraded ${\mathrm{CrBr}}_{3}$ flakes. Our study elucidates the effect of degradation on performance and the derived physical phenomena in devices based on air-sensitive 2D magnetic materials, offering practical guidance on the characterization and applications of 2D magnetic materials in ambient conditions.

## Data Availability

Not applicable.

## Supplementary Materials

The following supporting information can be downloaded at: https://www.mdpi.com/article/10.3390/ma15093007/s1, Figure S1: The magnetization of the CrBr_3_ bulk platelet as a function of in-plane magnetic field in the temperature regime from 3 to 100 K.; Figure S2: (a,b) Optical microscopy images of the CrBr_3_ flakes exposed to a stronger light irradiation in air for 0 min and 4 min, respectively.
