# Supplementary material for: Degradation Effect and Magnetoelectric Transport Properties in CrBr3 Devices
Source: Materials (Basel). 2022 Apr 21;15(9):3007. doi: 10.3390/ma15093007 (PMC9104061; doi:10.3390/ma15093007)
Supplement: Supplementary file 1 [file materials-15-03007-s001.zip › materials-1663975-supplementary.pdf]

Supplementary Materials

Degradation effect and magnetoelectric transport properties in $CrBr_3$ devices

Yanfei Wu[1], Mengyuan Zhu[1], Ruijie Zhao[1], Xinjie Liu[1], Jianxin Shen[1], He Huang[1], Shipeng Shen[2], Liyuan Zhang[1], Jingyan Zhang[1], Xinqi Zheng[1] and Shouguo Wang[1, *]

[1] Beijing Advanced Innovation Center for Materials Genome Engineering, School of Materials Science and Engineering, University of Science and Technology Beijing, Beijing 100083, China

[2] Institute of Advanced Materials, Beijing Normal University, Beijing 100875, China

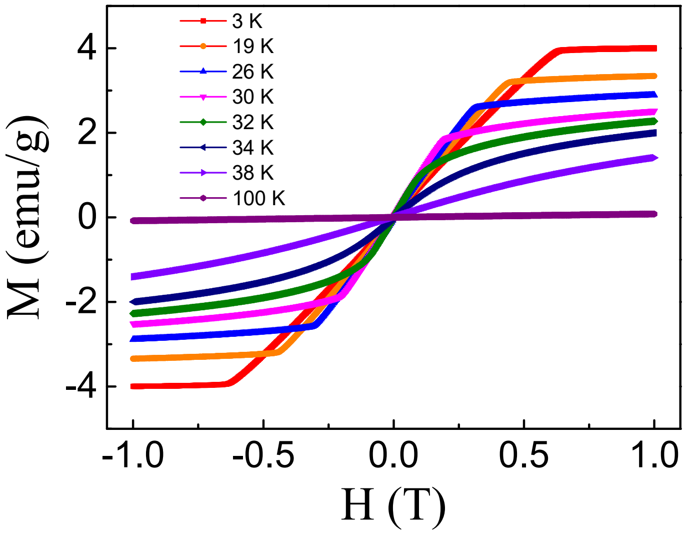

Figure S1. The magnetization of the $CrBr_3$ bulk platelet as a function of in-plane magnetic field in the temperature regime from 3 to 100 K.

As shown in Figure S1, M–H curves indicate that the ferromagnetic-paramagnetic phase transition temperature is about 34 K, nearly close to the value obtained by M–T curves (Figure 1b).

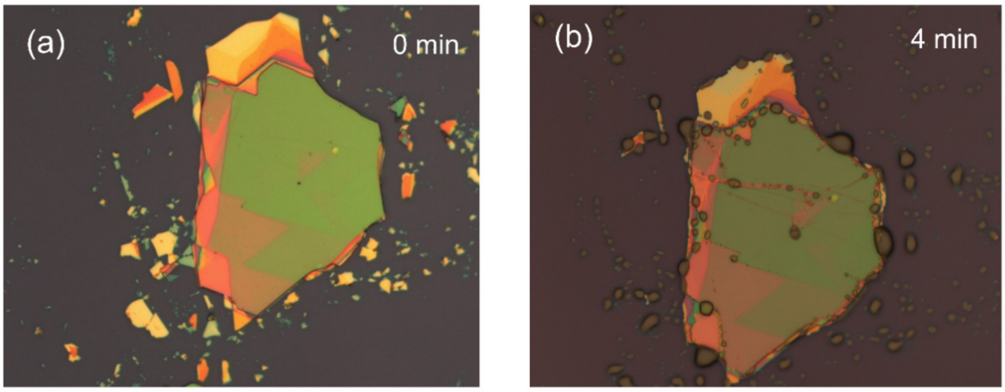

Figure S2. (a, b) Optical microscopy images of the $CrBr_3$ flakes exposed to a stronger light irradiation in air for 0 min and 4 min, respectively. The stronger light irradiation is provided by the light emitted from a 100× Objective in another optical microscope.

In Figure 3, the flakes were monitored by an optical microscope which provided light irradiation from a 50× Objective, with the distance of 20 mm between the objective and the flakes.

In addition, the degradation process for the flakes with different thicknesses were also monitored by another optical microscope in which a stronger light irradiation can be provided by the light emitted from a 100× Objective. The objective and the flakes are only a few millimeters apart, possibly resulting in higher temperature. Optical microscopy images of the degradation process in thin $CrBr_3$ flakes is shown in Figure 3g–3i. Figure S2 shows the optical microscopy images of thick $CrBr_3$ flakes exposed to the same strong light irradiation for 0 min and 4 min, respectively. The observations from Figure 3 and Figure S2 display the faster degradation with reduced size and thickness of $CrBr_3$ flakes. This implies that there is thickness-dependent degradation effect on magnetic properties or magnetoelectric transport behavior of 2D magnetic materials and their heterostructures.
